# Multifocal breast cancers are more prevalent in *BRCA2* versus *BRCA1* mutation carriers

**DOI:** 10.1002/cjp2.155

**Published:** 2020-02-05

**Authors:** Alan D McCrorie, Susannah Ashfield, Aislinn Begley, Colin Mcilmunn, Patrick J Morrison, Clinton Boyd, Bryony Eccles, Stephanie Greville‐Heygate, Ellen R Copson, Ramsey I Cutress, Diana M Eccles, Kienan I Savage, Stuart A McIntosh

**Affiliations:** ^1^ Centre for Cancer Research and Cell Biology Queen's University Belfast Belfast UK; ^2^ University of Cambridge School of Clinical Medicine Cambridge Biomedical Campus Cambridge UK; ^3^ Northern Ireland Regional Genetics Centre Belfast Health and Social Care Trust Belfast UK; ^4^ Institute of Pathology Royal Victoria Hospital Belfast UK; ^5^ Dorset Cancer Centre Poole General Hospital Dorset UK; ^6^ University of Southampton and University Hospital Southampton Southampton UK

**Keywords:** multifocal, breast cancer, *BRCA*, mutation, pathology, prevalence, epidemiology

## Abstract

Multifocal (MF)/multicentric (MC) breast cancer is generally considered to be where two or more breast tumours are present within the same breast, and is seen in ~10% of breast cancer cases. This study investigates the prevalence of multifocality/multicentricity in a cohort of *BRCA1/2* mutation carriers with breast cancer from Northern Ireland via cross‐sectional analysis. Data from 211 women with *BRCA1/2* mutations (*BRCA1*‐91, *BRCA2*‐120) and breast cancer were collected including age, tumour focality, size, type, grade and receptor profile. The prevalence of multifocality/multicentricity within this group was 25% but, within subgroups, prevalence amongst *BRCA2* carriers was more than double that of *BRCA1* carriers (*p* = 0.001). Women affected by MF/MC tumours had proportionately higher oestrogen receptor positivity (*p* = 0.001) and lower triple negativity (*p* = 0.004). These observations are likely to be driven by the higher *BRCA2* mutation prevalence observed within this cohort. The odds of a *BRCA2* carrier developing MF/MC cancer were almost four‐fold higher than a *BRCA1* carrier (odds ratio: 3.71, CI: 1.77–7.78, *p* = 0.001). These findings were subsequently validated in a second, large independent cohort of patients with *BRCA*‐associated breast cancers from a UK‐wide multicentre study. This confirmed a significantly higher prevalence of MF/MC tumours amongst *BRCA2* mutation carriers compared with *BRCA1* mutation carriers. This has important implications for clinicians involved in the treatment of *BRCA2*‐associated breast cancer, both in the diagnostic process, in ensuring that tumour focality is adequately assessed to facilitate treatment decision‐making, and for breast surgeons, particularly if breast conserving surgery is being considered as a treatment option for these patients.

## Introduction

A large meta‐analysis of 22 studies, including over 67 000 women, estimated the prevalence of multifocal (MF) breast cancer to be 9.5% 1. Although multifocality does not appear to be an independent predictor of outcome in breast cancer, the sum of the invasive foci in MF disease may be associated with reduced disease‐free survival, when compared with unifocal tumours 2, 3, 4. Moreover, treatments offered for MF breast cancer vary widely, with some women undergoing multiple breast conserving procedures and others mastectomy, with no clear treatment guidelines 5.

Historically, the definitions of MF and multicentric (MC) breast cancer have varied. MF cancers have been defined as two or more distinct invasive breast carcinomas within the same breast quadrant, whereas MC disease has been defined as separate tumours in different breast quadrants. Studies have suggested that, in cases of both MF and MC disease, tumours may either share or be of independent clonal origin 6, 7, 8, 9. Furthermore, published data suggest that MF and MC disease may have different patterns of behaviour clinically 10. Due to these conflicting definitions and the lack of clarity on whether these entities represent the same disease process, for the pragmatic purposes of this retrospective study and to avoid confusion, we have considered MF and MC tumours together.

*BRCA1* and *BRCA2* are tumour suppressor genes located on chromosomes 17 and 13 respectively. They encode proteins involved in the cellular DNA damage response pathway, particularly DNA double strand break repair 11. Germline mutations in these genes predispose female carriers to a significantly increased risk of breast and ovarian cancer, with up to 80% lifetime risk of breast cancer. Given this elevated breast cancer risk, we hypothesised that these women may be more likely than non‐mutation carriers to develop MF disease. Surprisingly, despite biological plausibility for the existence of an association between *BRCA1/2* mutations and MF/MC tumours, at the time of writing, there were no studies investigating this. Therefore, this study aimed to investigate the prevalence of MF/MC breast cancer in *BRCA1/2* mutation carriers, with exploration of the clinicopathological characteristics of all tumours occurring in these patients.

## Materials and methods

**Figure 1:**
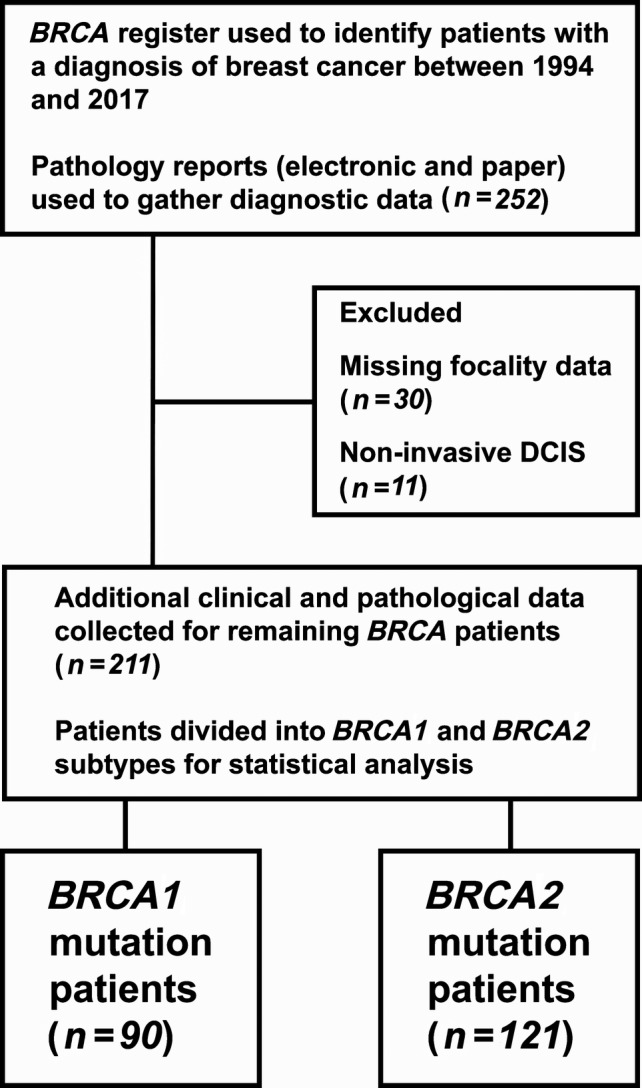
Participant flow diagram showing inclusions/exclusions.

Data from 252 women with a known pathogenic germline *BRCA1/2* mutation diagnosed with breast cancer (1994–2017) were retrospectively extracted from a database containing all known female *BRCA1/2* mutation carriers in Northern Ireland (Figure 1). Information about histological tumour type and focality (unifocal or MF) was extracted from pathology records for 211 women, with 41 patients excluded due to missing focality information (*n* = 30), or because of a diagnosis of ductal carcinoma *in situ* without invasion (*n* = 11). Additional clinicopathological data were collected, including age at initial cancer diagnosis, tumour grade and size, hormone receptor status, HER2 status, nodal involvement, presence/absence of other primary cancer. Outcome data was collected from electronic hospital records, and cause of death ascertained. Data were entered into Microsoft Excel® (Redmond, Washington, USA) for stratification and calculation of prevalence. Twenty‐three randomly selected cases (10%) underwent review of the original diagnostic slides by an independent pathologist for validation of multifocality/multicentricity reporting.

For validation of the findings in the Northern Ireland patient cohort, a second cohort of breast cancer patients with known germline *BRCA1/2* mutations was identified. The POSH (Prospective Outcomes in Sporadic versus Hereditary breast cancer) study recruited young women (aged 18–40) diagnosed with primary breast cancer in the United Kingdom between 2000 and 2008 12. The study methodology (including genotyping methods) and outcomes have previously been reported 13. Data on tumour focality/centricity in the POSH study patients was obtained from medical records from participating centres.

Data were analysed using SPSS®. Heterogeneity of clinicopathological characteristics between those diagnosed with unifocal disease and those diagnosed with MF/MC disease were compared using χ^2^. Mean age and tumour size between groups was compared using the *t*‐test. Binary logistic regression was performed to calculate the unadjusted odds ratio (OR) of developing MF disease in patients with *BRCA2*‐associated breast cancer versus those with *BRCA1*‐associated breast cancer. Thereafter, adjusted OR was calculated using a manually controlled backward stepwise elimination approach 14. Potentially confounding variables with a biological association to breast cancer were entered into the regression model and sequentially removed until only those with statistical significance remained. Survival estimates were carried out using the Kaplan–Meier method. A *P* value of <0.05 indicates significance at the 95% confidence interval throughout. Institutional approval was granted by the Belfast Health and Social Care Trust (Ref: 5805). Ethical approval for the POSH study was granted in 2000 (MREC 00/6/69).

## Results

### Northern Ireland *BRCA1/2* cohort

Of the 211 *BRCA1/2* carriers diagnosed with breast cancer (with MF/MC information available) in Northern Ireland between 1994 and 2017, 90 (42.7%) women had a *BRCA1* mutation and 121 (57.3%) a *BRCA2* mutation. Mean age at diagnosis was 45 years (range: 25–77 years) with a lower mean age at diagnosis for MF/MC tumours compared with unifocal tumours (43 versus 46 years) (*p* = 0.109). Mean tumour size was 24 mm (range: 2–150 mm) with no significant difference in mean size between the largest MF/MC tumour foci and unifocal tumours (24.8 mm versus 23.2 mm) (*p* = 0.587). There were 52 diagnoses of MF/MC disease and 159 diagnoses of unifocal disease. The prevalence of MF/MC disease was 13.3% in *BRCA1* mutation carriers and 33.1% in *BRCA2* mutation carriers. Therefore, the prevalence of MF/MC disease in *BRCA2* carriers was 2.5‐fold greater than in *BRCA1* carriers (*p* = 0.001). Clinicopathological findings are documented in Table 1. The majority of MF/MC and unifocal tumours were invasive ductal carcinomas (86.5 and 96.2% respectively), grade III (73.6 and 63.5% respectively), and HER2‐negative (75.0 and 73.6% respectively). Additionally, *BRCA1/2* carriers with MF/MC disease were more likely to be oestrogen receptor positive than negative (75.0% versus 45.9%) (*p* = 0.001). Furthermore, it is known that invasive lobular carcinoma (ILC) is seen more frequently in *BRCA2* than *BRCA1* mutation carriers 15. We therefore excluded the nine cases of ILC in this cohort (seven *BRCA2* mutation carriers and two *BRCA1* mutation carriers), and repeated the analysis including invasive ductal carcinoma alone, showing that multifocality/multicentricity remained significantly higher in *BRCA2* versus *BRCA1* mutation carriers when cases of ILC were excluded (*p* = 0.001).

**Table 1:** Clinical and pathological characteristics of *BRCA1/2* mutation carriers within the Northern Ireland patient cohort

Clinical and pathological features of breast cancers		Multifocality *n* (%)	Unifocality *n* (%)	*P* value*
*BRCA* mutation	*BRCA1*	12 (13.3)	78 (86.7)	0.001
	*BRCA2*	40 (33.1)	81 (66.9)	
Age at first diagnosis	<40 years	23 (32.9)	47 (67.1)	0.039
	≥40 years	29 (20.6)	112 (79.4)	
Tumour subtype	Invasive ductal	45 (22.7)	153 (77.3)	0.011
	Invasive lobular	6 (66.7)	3 (33.3)	
	Other	1 (25.0)	3 (75.0)	
Tumour grade	I	3 (42.9)	4 (57.1)	0.460
	II	15 (30.0)	35 (70.0)	
	III	33 (22.0)	117 (78.0)	
	Unknown	1 (25.0)	3 (75.0)	
Oestrogen receptor	Positive	39 (34.8)	73 (65.2)	0.001
	Negative	12 (12.9)	81 (87.1)	
	Unknown	1 (16.7)	5 (83.3)	
HER2 status	Positive	3 (27.3)	8 (72.7)	0.933
	Negative	39 (25.0)	117 (75.0)	
	Unknown	10 (22.7)	34 (77.3)	
Triple negativity	Yes	7 (12.5)	49 (87.5)	0.004
	No	30 (36.1)	53 (63.9)	
	Unknown	15 (20.8)	57 (79.2)	
Lymph node involvement	Yes	26 (33.3)	52 (66.7)	0.072
	No	25 (20.0)	100 (80.0)	
	Unknown	1 (12.5)	7 (87.5)	
Presence of other primary cancer	Yes	9 (23.7)	29 (76.3)	0.957
	No	42 (25.0)	126 (75.0)	
	Unknown	1 (20.0)	4 (80.0)	

* Pearson's χ^2^ where *p* < 0.05 indicates significance.

Of the 52 women diagnosed with MF/MC disease, 23.1% (*n* = 12) had a *BRCA1* mutation and 76.9% (*n* = 40) a *BRCA2* mutation. 50% (*n* = 6) of women with a *BRCA1* mutation were oestrogen receptor positive whilst 82.5% (*n* = 33) women with a *BRCA2* mutation were oestrogen receptor positive (*p* = 0.039). See supplementary material, Table S1. The unadjusted odds of breast cancer being MF/MC in *BRCA2* mutation carriers were 3.2 times greater than in *BRCA1* mutation carriers (CI: 1.57–6.57, *p* = 0.001). Age was found to be a significant confounding factor in logistic regression (CI: 0.22–0.85, *p* = 0.015). After adjusting for age, the odds of a *BRCA2* mutation carrier developing MF/MC breast cancer were 3.7‐fold greater than in *BRCA1* mutation carriers (CI: 1.77–7.78, *p* = 0.001) (Table 2).

**Table 2:** Odds of cancer being MF in patients with *BRCA2* versus *BRCA1* mutation

Variable	Odds ratio (95% CI)	*P* value
*BRCA* status*	3.21 (1.57–6.57)	0.001
*BRCA* status†	3.71 (1.77–7.78)	0.001
*BRCA* status‡	5.79 (3.31–10.12)	<0.001

* Unadjusted OR in the Northern Ireland cohort.

† Odds ratio in the Northern Ireland cohort adjusted for age (≥40 years versus <40 years).

‡ Unadjusted OR in the POSH study cohort.

**Figure 2:**
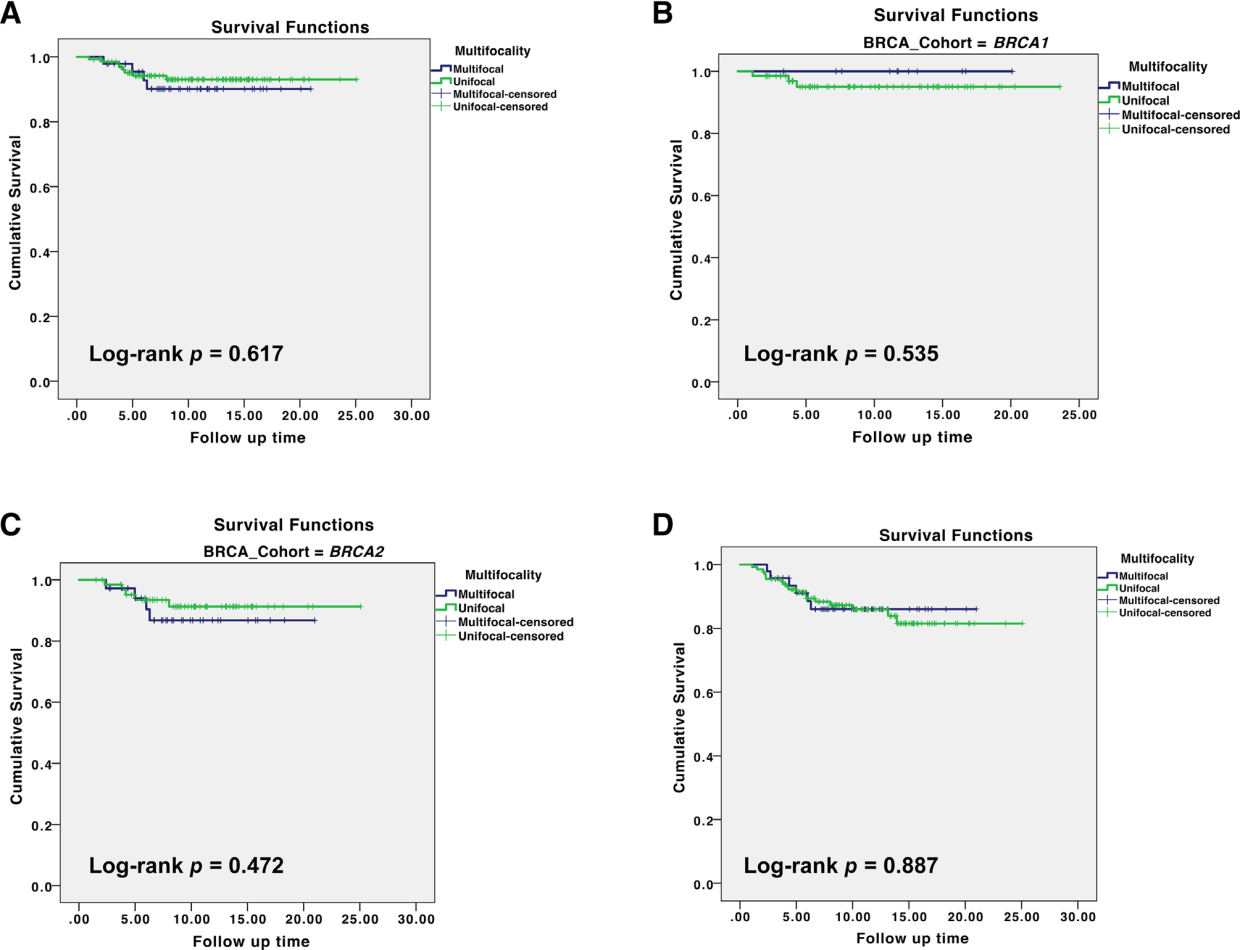
Survival outcomes in the Northern Ireland patient cohort. (A) MF/MC disease versus unifocal disease, breast cancer specific mortality, all patients (*BRCA1/2*). (B) MF/MC disease versus unifocal disease, breast cancer specific mortality in *BRCA1* mutation carriers. (C) MF/MC disease versus unifocal disease, breast cancer specific survival in *BRCA2* mutation carriers. (D) MF/MC versus unifocal disease, all‐cause mortality in all patients (*BRCA1/2*).

At a median follow‐up of 9.5 years for the cohort of Northern Irish patients, there was no breast cancer‐specific survival difference between women with MF/MC versus unifocal disease (log‐rank *p* = 0.617), and when adjusted for *BRCA* mutation status this remained non‐significant (log‐rank *p* = 0.775) (Figure 2A). Similarly, there was no difference in survival between MF/MC or unifocal tumours in *BRCA1* mutation carriers (Figure 2B) or *BRCA2* mutation carriers (Figure 2C), nor was there a difference in all‐cause survival between women with unifocal versus MF/MC disease (Figure 2D).

### POSH study *BRCA1/2* cohort

There were 338 germline *BRCA* mutation carriers in the POSH study breast cancer cohort; focality data were missing in 37 cases, leaving 180 women with a *BRCA1* mutation and 121 with a *BRCA2* mutation for analysis. There were 81 diagnoses of MF/MC disease and 220 diagnoses of unifocal disease. Clinicopathological findings in the POSH cohort are detailed in Table 3. Mean age of diagnosis was 34 years, with no difference seen in the age at diagnosis for MF/MC tumours versus unifocal tumours (35 versus 34 years). MF/MC breast cancer was identified in 26.9% of *BRCA1/2* mutation carriers who developed breast cancer. The prevalence of MF/MC disease was 13.3% amongst *BRCA1* mutation carriers diagnosed with breast cancer, and 47.1% amongst *BRCA2* mutation carriers diagnosed with breast cancer. Therefore, the prevalence of MF/MC disease in *BRCA2* mutation carriers was 3.5‐fold greater than in *BRCA1* mutation carriers in this independent cohort of *BRCA1/2* carriers (*p* < 0.001).

**Table 3:** Clinical and pathological characteristics of *BRCA1/2* mutation carrier patients diagnosed with breast cancers within the POSH dataset (2000–2008)

Clinical and pathological features of breast cancer		Multifocality *n* (%)	Unifocality *n* (%)	*P* value*
*BRCA* mutation	*BRCA1*	24 (13.3)	156 (86.7)	<0.001
	*BRCA2*	57 (47.1)	64 (52.9)	
Tumour grade	I	0 (0.0)	2 (100.0)	0.009
	II	23 (46.0)	27 (54.0)	
	III	57 (23.3)	188 (76.6)	
	Unknown	1 (25.0)	3 (75.0)	
Oestrogen receptor	Positive	60 (39.7)	91 (60.3)	<0.001
	Negative	21 (14.0)	129 (86.0)	
HER2 status	Positive	8 (40.0)	12 (60.0)	0.382
	Negative	65 (26.2)	183 (73.8)	
	Unknown	8 (24.2)	25 (75.8)	
Triple negativity	Yes	15 (13.9)	93 (86.1)	<0.001
	No	63 (37.3)	106 (62.7)	
	Unknown	8 (24.2)	21 (87.5)	
Lymph node involvement	Yes	55 (39.6)	84 (60.4)	0.009
	No	25 (15.5)	136 (84.5)	
	Unknown	1 (100)	0 (0.0)	

* Pearson's χ^2^ where *p* < 0.05 indicates significance.

*BRCA1/2* mutation carriers with MF/MC disease were more likely to be oestrogen receptor positive (74.1%) than those with unifocal disease (41.4%). This difference in proportion was significant (*p* < 0.001). Similarly, *BRCA1/2* mutation carriers with MF/MC disease were less likely to be triple receptor negative (18.5%) compared to those with unifocal disease (42.3%). This was also significant (*p* < 0.001). When data from women who developed MF/MC breast cancer were analysed in isolation, the prevalence of oestrogen receptor positivity was 85% amongst *BRCA2* mutation carriers but only 15% in *BRCA1* mutation carriers. This difference in proportion was significant (*p* < 0.001) (see supplementary material, Table S2).

The unadjusted odds (in binary logistic regression analysis) of a breast cancer being MF/MC in *BRCA2* mutation carriers was 5.8 times greater than in a *BRCA1* mutation carrier who developed breast cancer (CI: 3.31–10.12) (*p* < 0.001) (Table 2). Adjustment for oestrogen receptor status gave odds of a *BRCA2* mutation carrier developing MF/MC breast cancer 4.2 times greater than a *BRCA1* mutation carrier (CI: 2.12–8.19) (*p* < 0.001). A similar reduction in the magnitude of the OR was observed in our dataset when the analysis was adjusted for oestrogen receptor status.

## Discussion

A systematic review of sporadic MF/MC breast cancer conducted by Vera‐Badillo *et al* included 22 studies encompassing 67 557 women. This study calculated a prevalence of 9.5% amongst women with sporadic breast cancer (*BRCA* status unknown) 1. It should also be noted that this meta‐analysis only includes women with early stage breast cancer, and only includes studies which provided survival outcome data, so it is possible that this is not truly representative of the incidence of MF/MC disease in the general population. Nevertheless, and in contrast, the prevalence of multifocality/multicentricity in the Northern Ireland cohort of 211 *BRCA1/2* mutation carriers was 24.6%, more than double that reported by Vera‐Badillo *et al* for sporadic breast cancer 1. This finding is supported by a strikingly similar prevalence of 26.9% in the larger cohort of *BRCA1/2* mutation carriers from the multicentre POSH study.

Our study found that the prevalence of multifocality/multicentricity in *BRCA2* mutation carriers was at least double that in *BRCA1* mutation carriers in both reported patient cohorts – a finding mirrored in a small‐scale study by Bergthorsson *et al* 16. The odds of a woman who had developed breast cancer exhibiting MF/MC disease were over three times greater if she had a *BRCA2* mutation compared to a *BRCA1* mutation. This rose to an almost four‐fold increase in odds of a *BRCA2* carrier developing MF/MC disease once the effect of age at diagnosis was taken into account.

Women diagnosed with MF/MC breast cancer were proportionately more likely to be oestrogen receptor positive and had a lower prevalence of triple receptor negativity in both the Northern Ireland and the POSH study patients. These findings are in keeping with numerous studies documenting significantly higher rates of oestrogen receptor positivity amongst *BRCA2* carriers compared with *BRCA1* mutation carriers. Therefore, it is unlikely that oestrogen signalling itself drives MF/MC disease 17, 18. Indeed, the large‐scale meta‐analysis described earlier found no association between ER status and sporadic MF/MC breast cancer, suggesting that ER activity does not play a role in the specific development of MF/MC disease 1. Similarly, although ILC was seen more commonly in *BRCA2* mutation carriers than in *BRCA1* carriers in the Northern Ireland patient cohort, the significant increase in prevalence of MF/MC disease in *BRCA2* carriers persisted even when ILC cases were excluded from the analysis, suggesting that it is not the lobular phenotype which drives the increased prevalence of multifocality/multicentricity.

Precisely why *BRCA2* carriers are more likely to develop MF/MC disease than *BRCA1* carriers is unclear. Recent evidence suggests that *BRCA1*‐related breast cancer is driven by aberrant RANK/RANKL signalling in *BRCA1* heterozygous luminal progenitor cells, coupled with increased DNA damage/defective DNA repair in these cells, resulting in development of basal breast cancers 19. In contrast, this has not been reported in *BRCA2* carriers, who predominately develop luminal breast cancer. Additionally, *BRCA2*'s predominant reported function is its direct role in homologous recombination‐mediated double strand break repair 20. Clearly, a better understanding of molecular and genetic processes resulting in the development of basal and luminal breast cancers at the single cell level is required. Moreover, given the apparent predominant development of synchronous but distinct cancers in *BRCA2* mutation carriers, the contribution of genomic instability at a single cell level also needs to be investigated. Finally, given recent data demonstrating activation of cell intrinsic innate immune responses to the loss of *BRCA1/2*, the role of early immunoediting in control of tumours in *BRCA1* versus *BRCA2* carriers needs to be investigated 21.

This study is strengthened by relatively complete data. Data for all 211 women from Northern Ireland are complete with regard to tumour focality, *BRCA* mutation type, age at diagnosis, and tumour type. Furthermore, information about tumour grade, oestrogen receptor status, lymph node involvement, and presence or absence of other primary tumours are all in excess of 96% complete. The considerable quantity of missing HER2 status data reflects the fact that HER2 testing was not routinely carried out at the time of diagnosis for many of these women 22. Additionally, the presence of a large, independent cohort of *BRCA1/2* mutation carriers in the validation cohort provides strong evidence to support the increased prevalence of MF/MC tumours in *BRCA2* mutation carriers.

With respect to the long‐term outcomes in the Northern Ireland cohort of patients, no difference in outcomes was noted between MF/MC and unifocal tumours, even when adjusted for *BRCA* mutation status, although these data need to be interpreted with caution due to the small number of deaths in the two groups. Other groups have reported worse 10 year survival in *BRCA1* mutation carriers as compared with *BRCA2* carriers, ascribing this difference to tumour biology 23. However, the cohort of young patients from the POSH study, which form the validation cohort for this study, did not demonstrate a significant difference in overall survival between either *BRCA1* or *BRCA2* mutation carriers and non‐mutation carriers, despite showing a similar increase in multifocality/multicentricity prevalence in *BRCA2* mutation carriers 13. Taken together, these data support the contention that multifocality/multicentricity is not an independent prognostic factor in breast cancer. Due to the retrospective nature of this series, it was not possible to add together the tumour diameters of individual foci as carried out by Fushimi *et al* 2. Although Fushimi *et al* suggested that doing so may predict for outcome, this may simply be due to the fact that it reflects a higher burden of tumour in these patients rather than being a function of multifocality/multicentricity *per se*.

There are necessarily limitations to a retrospective review of MF/MC breast cancer, as much of the macroscopic pathological information available at the time of initial surgery is no longer available at retrospective slide review. In this study, the assumption was made that the original diagnosis of MF/MC disease (as made by the reporting pathologist with all the macroscopic and microscopic information to hand) was accurate, and a slide review was carried out to determine whether there were any features to suggest that this original diagnosis was incorrect. Indeed, in the 10% of cases where slides were reviewed there was no evidence that the diagnosis of MF/MC cancer was incorrect, and no cases were excluded on the basis of this review. Furthermore, data on the number of tumour foci and the intervening distance between foci in each case, and their morphological similarities/differences, were not available. Biomarker status (ER/PR/HER2) was also not available for individual tumour foci, as this was generally not assessed on all tumour foci, meaning that it is not possible to comment on the morphological nature of the MF/MC disease in these patients. These limitations are applicable to both the Northern Ireland and POSH study patient cohorts.

In conclusion, we report a higher than anticipated prevalence of multifocality/multicentricity amongst female *BRCA1/2* mutation carriers diagnosed with breast cancer. This finding was seen in a cohort of patients from Northern Ireland and is validated in the independent cohort of *BRCA1/2* mutation carriers from the POSH study. Our data also suggest that multifocality/multicentricity is more common in *BRCA2*‐associated breast cancer. Those with MF/MC disease were more likely to be younger at diagnosis, and more likely to be oestrogen receptor positive than those with unifocal disease. These findings have important implications for clinicians involved in the care of patients with *BRCA‐*associated breast cancer, who will need to ensure that *BRCA2‐*associated tumour focality is thoroughly assessed during the diagnostic process. Furthermore, where breast conserving surgery is being considered as a treatment option for these patients, surgeons need to be aware of the increased incidence of multifocality and plan surgery accordingly, to ensure complete excision at one operation and minimise the consequences associated with re‐operation due to involved margins. Finally, further studies are required to establish the underlying mechanistic basis for these findings.

## Author contributions statement

SAM and KIS conceived the study. ADM, SA, AB and PJM collected data. BE, SG‐H, ERC, RIC and DME provided data for the POSH cohort. ADM, SA, CM and AB performed statistical analysis. Verification was performed by PJM and CB. The manuscript was drafted and critically revised by all authors; all authors have approved the final version of the manuscript.

## Supporting information


**Table S1.** Oestrogen receptor status of the Northern Ireland cohort of female *BRCA1/2* mutation carriers diagnosed with multifocal breast cancer between 1994 and 2017
**Table S2.** Oestrogen receptor status of the POSH study cohort of female *BRCA1/2* mutation carriers
