# Supplementary material for: Multifocal breast cancers are more prevalent in BRCA2 versus BRCA1 mutation carriers
Source: J Pathol Clin Res. 2020 Feb 5;6(2):146–53. doi: 10.1002/cjp2.155 (PMC7164372; doi:10.1002/cjp2.155)
Supplement: Supplementary file 1 — Table S1. Oestrogen receptor status of the Northern Ireland cohort of female BRCA1/2 mutation carriers diagnosed with multifocal breast cancer between 1994 and 2017 Table S2. Oestrogen receptor status of the POSH study cohort of female BRCA1/2 mutation carriers [file CJP2-6-146-s001.docx]

**Multifocal breast cancers are more prevalent in *BRCA2* versus *BRCA1* mutation carriers**

McRorie AD *et al. J Pathol Clin Res* DOI: 10.1002/cjp2.155

**Table S1:** Oestrogen receptor status of the Northern Ireland cohort of female *BRCA1/2* mutation carriers. *Pearson’s χ^2^ where p < 0.05 indicates significance.

| **Women with pathologically confirmed multifocal breast cancer** | | ***BRCA1* mutation**  **N (%)** | ***BRCA2* mutation**  **N (%)** | **P value**  **(*)** |
| --- | --- | --- | --- | --- |
| Oestrogen receptor status | Positive  Negative  Missing | 6 (15.4)  6 (50.0)  0 (0.0) | 33 (84.6)  6 (50.0)  1 (100.0) | 0.039 |

**Table S2:** Oestrogen receptor status of the POSH study cohort of female *BRCA1/2* mutation carriers. *Pearson’s χ^2^ where p < 0.05 indicates significance.

| **Women with pathologically confirmed multifocal breast cancer** | | ***BRCA1* mutation**  **N (%)** | ***BRCA2* mutation**  **N (%)** | **P value**  **(*)** |
| --- | --- | --- | --- | --- |
| Oestrogen receptor status | Positive  Negative  Missing | 9 (37.5)  15 (71.4)  0 (0.0) | 51 (85.0)  6 (28.6)  0 (0.0) | < 0.001 |
